# Unexpected cause of esophageal obstruction due to accidental use of traditional medicine in a critically ill patient fed through naso-gastric tube

**DOI:** 10.4103/0972-5229.74178

**Published:** 2010

**Authors:** Qutaiba Amir Tawfic, Pradipta Bhakta, Rajini Kausalya

**Affiliations:** Sultan Qaboos University Hospital, Muscat, Oman

Dear Editor,

Enteral nutrition is an integral part of management of patients in ICU.[1] Use of enteral nutrition through a nasogastric tube (NGT) can be associated with esophageal obstruction and bezoars formation.[1,2] Here, we report such a case of esophageal obstruction and bezoars formation due to inadvertent use of traditional herbal medicine.

A 39-year-old patient without any history of medical problem presented with repeated convulsions, with 4 days history of fever and headache. She was treated with all possible anticonvulsants, but did not respond initially and was labeled as resistant status epilepticus. During a brief seizure free period, Magnetic Resonance Imaging (MRI) of the brain was tried, but it failed due to movement artifacts. As she was desaturating, she was shifted to the ICU and was put on a ventilator after endotracheal intubation. She was started on a high dose of sedatives and multiple anticonvulsants for control of seizures. Post-intubation chest X-ray showed features of aspiration pneumonitis. Continuous electro-encephalogram (EEG) monitoring was started. Computerized tomography (CT) scan of brain and diagnostic lumbar puncture were done, but these reports came as normal. So, a provisional diagnosis of Herpes encephalitis was made and she was started on acyclovir. NGT was inserted and position was confirmed clinically as well as radiologically. NG feeding was started after that. Seizure could be controlled after 8 days with heavy doses of anticonvulsants. Repeat MRI and lumber puncture also came normal. On the 9^th^ day, we noticed obstruction of the NG tube. After all tests were done, NGT was pulled out with some force. Numerous attempts to reinsert a new NGT failed due to resistance against forward insertion. She was then referred to a Gastroenterologist for flexible upper gastrointestinal endoscopy (UGIE). Endoscopy revealed white-chalky solid and semisolid like concretions extending from upper esophagus. Further introduction of endoscope was only possible after several esophageal wash outs. Stomach and duodenum were found to be normal. Naso-duodenal feeding tube was subsequently inserted and feeding resumed without any problem. While we were explaining the condition, relatives revealed that they themselves gave some traditional herbal medicine orally for control of ongoing uncontrolled seizure. We concluded that esophageal obstruction was caused by that traditional medicine. Her family refused to reveal the name of the medicine. After 21 days, we could wean off all sedatives and she was started on oral antiepileptics. She was ultimately tracheostomized and discharged from ICU after 40 days, as she developed aspiration pneumonia and gram negative sepsis. Later, she was discharged home.

It is believed that NGT feeding formulas, after reacting with gastric acid as well as medicines, can result in formation of a solid mass (bezoar).[2] Presence of NGT or endotracheal tube increases reflux of gastric acid or enteral feed which can react with medicines to form a bezoar in the esophagus.[2–4] It is possible that the orally administered traditional herbal medicine might have reached the stomach or lower esophagus and reacted with gastric acid or regurgitated enteral feed to form a solid mass which obstructed the NGT and esophagus.

Bezoars can be mainly of five types, namely, phytobezoars (resulting from undigested vegetables or fruits, commonest of all), pharmacobezoars (resulting from undigested medicines), trichobezoras (due to ingestion of hairs), lactobezoars (seen in neonates due to ingestion of milk), and foreign body bezoars.[3–6] Gastric and/or esophageal pathologies like dysmotility, previous operations, diabetes, hypothyroidism and psychological illness have been reported to predispose bezoar formation.[3,4,6] UGIE has been recommended for diagnosis as well as removal of the bezoras by fragmentation and washing.[3–6] Most pharmacobezoars are reported to form in the stomach or small intestine.[3–6] Reverse migration of gastric content and reaction with medicines can very rarely cause esophageal bezoar formation.[2–4] Pharmacobezoars are mainly reported to form due to ingestion of enteric coated aspirin, antacids, sucralfate, bulk laxatives, iron, nifedipine.[2,3–6] Very rarely, it has been reported to occur from ingestion of traditional herbal medicines.[4–6] All of them are reported to occur in the stomach or small intestine. Literature search did not reveal any report of esophageal bezoar formation due to inadvertent ingestion of herbal medicine.

Administration of any traditional medicine to an unconscious patient by attendants can lead to life-threatening esophageal obstruction and there should be a specific guideline to prevent and control this problem.
